# Supplementary material for: CIPHER: An end-to-end framework for designing optimized aggregated spatial transcriptomics experiments
Source: PLoS Comput Biol. 2026 Jun 4;22(6):e1014362. doi: 10.1371/journal.pcbi.1014362 (PMC13252831; doi:10.1371/journal.pcbi.1014362)
Supplement: Attachment — Submitted filename: Response to reviewers.pdf [file pcbi.1014362.s005.pdf]

Dear Drs. Wan and Mendes,

Thank you for the feedback on our manuscript and for the opportunity to submit a revised version.

In the revision, we addressed the main issues highlighted by the reviewers, as detailed in our response letter. This included additional benchmarking and clarification of the benchmarking already performed, and revisions to the evaluation framework. We also revised the noise model and expanded the robustness analyses, including parameter sensitivity analyses, controlled stress tests, and practical guidance for hyperparameter tuning.

In addition, we substantially revised the Methods section to provide a more complete and precise description of the approach. We now include the full loss-function equations, clearer definitions of all evaluation metrics, and additional justification of preprocessing choices, including CP100k normalization.

We appreciate the reviewers' careful evaluation and believe these revisions have substantially strengthened the manuscript.

In the point-by-point response below, we kept the reviewer's text in black, used red for our response, and highlighted revisions in blue. We only included revision text when space allowed.

Sincerely,
Roy

Reviewers' comments:

Reviewer #1:

Here, Hemminger et al. proposed CIPHER, an end-to-end neural network framework that jointly optimizes probe encoding design and cell-type decoding for aggregate spatial transcriptomics. The key innovation is incorporating experimental constraints (probe budget, signal brightness, dynamic range) directly into the loss function, enabling end-to-end optimization that balances classification accuracy with measurement robustness. Using the spatial transcriptomics datasets under three contexts, the authors demonstrated that accuracy-only optimization yields encodings that are fragile under realistic noise, while CIPHER's multi-objective approach achieves robust cell-type separation with minimal accuracy trade-off. The framework addresses an important gap between in silico optimization and in situ measurement feasibility. I have the following major concerns that need to be revised before I will be able to recommend the manuscript for publication:

We thank the reviewer for the careful evaluation of our manuscript and for the helpful summary of the main contribution of this work. We appreciate the reviewer's recognition that CIPHER addresses an important gap between in silico optimization and experimental feasibility in aggregate spatial transcriptomics. We also appreciate the reviewer's acknowledgment of the central idea of jointly optimizing encoding design and decoding under explicit measurement constraints. The comments below were useful in identifying places where the manuscript required stronger validation, clearer explanation, and additional methodological detail, and we have revised the manuscript accordingly.

Major Comments:

CIPHER is a supervised learning method utilizing cell-type labels, whereas DPNMF is unsupervised. Comparing exclusively against an unsupervised baseline is insufficient to demonstrate CIPHER's advantages. The authors should include comparisons with other supervised approaches, such as linear discriminant analysis on marker genes, supervised PCA, or other task-aware dimensionality reduction methods.

We completely agree with the reviewer that CIPHER should be compared with a supervised method, which is why we chose DPNMF, a supervised variant of NMF. We corrected the type, clarifying that DPNMF is supervised, and added the missing clarification text and citations from the original DPNMF manuscript.

We updated the text to fix the mistakes and justify the choice of DPNMF:

We compared CIPHER to DPNMF, a supervised discriminant non-negative matrix factorization approach previously used in ATLAS, using matched dimensionality and the same scRNA-seq reference \cite{guan2013discriminant,song2021scpnmf}. Similar to CIPHER, DPNMF uses a supervised approach to identify latent dimensions and shares other important characteristics with CIPHER, such as sparsity and non-negativity. Furthermore, DPNMF was used in prior experimental work that uses transcriptional signatures, making it the appropriate comparison to CIPHER.

The loss function weights are presented as fixed defaults without robustness analysis. Please provide sensitivity analysis showing how performance varies across weight configurations, and offer practical guidance for users on how to adjust hyperparameters for different tissues or experimental setups.

We now include a new figure (Figure 5) that performed sensitivity analysis for loss function weights. The figure shows that the three main evaluation metrics are not very sensitive to changes in the loss weights. Saying that, the CIPHER code does use specific targets in the optimization, which are important as shown in Figure 4, especially brightness. To provide more guidelines on how to chose these targets, we added a section to the Method (Selection of Target Values) that explains in detail the conversion of experimental poarameters related to the microscope and data acquisition setup and how they are translated into the loss targets used here. We also added the following text to the discussion section to provide more high-level guidelines that would supplement the details provided in the Methods section:

These results also suggest a practical interpretation for applying CIPHER across tissues and assay regimes. If designs achieve good nominal accuracy but poor robustness, increase separation and dynamic-range targets rather than decoder complexity. If bit intensities are systematically weak, increase brightness and probe-budget targets jointly. If performance saturates early with excess measurement capacity, reduce the number of bits or relax brightness targets.

The noise model is oversimplified. Training uses only uniform additive noise [0, 1e4], yet real experiments exhibit gene-specific probe efficiency variations, spatially structured background fluorescence, and even channel crosstalk. The authors should either fit a noise model from real MERFISH data, or at minimum incorporate gene-specific multiplicative noise to better approximate experimental conditions.

We thank the reviewer for this point. We agree that the original fixed additive perturbation was too limited as a model of experimental variability. In the revised manuscript, we now distinguish between the simple training-time noise used as augmentation and a separate post hoc robustness benchmark. Specifically, we retained additive latent-space background noise during training, but replaced the evaluation stress test with a signal-dependent multiplicative noise model calibrated to match the previous perturbation scale at the median bit intensity of each bit. We updated the robustness analysis accordingly and now describe both the rationale and the limitation of this simplified training-time noise model explicitly in the Methods and Discussion.

Minor Comments:

Definitions of evaluation metrics (Accuracy, Separation, and Dynamic Fold) are incomplete. Please provide explicit mathematical definitions in the corresponding section.

All evaluation metrics are now defined in the Evaluation Metrics Methods section.

Figure 5 requires clarification: (a) correct "seperation" to "separation" in axis labels; (b) specify axis labels as "cell-type separation" (y-axis) and "cell-type count" (x-axis) for better understanding of performance; (c) explain what the numerical values represent in panel 5b.

Figure updated.

In Figure 6, indicate which region of panel 6c corresponds to the zoomed view in panel 6d.

Figure updated.

Figure 7 captions do not match the panel descriptions in the main text. Please reconcile.

Fixed.

Reviewer #2:

The authors described CIPHER, which proposes an integrative end-to-end framework to compress spatial transcriptomic data by learning an assay-aware gene-to-signature encoding. The model incorporates realistic constraints (e.g., probe budget, noise, dynamic range) directly into the loss function with multiple terms to optimize together. This aims to produce compressed measurements that are both informative for cell-identity separation and feasible to measure experimentally. While promising, the current formulation may be specialized per experimental design/tissue and sensitive to loss term contribution, potentially limiting its broader utility for discovery-oriented biology.

We thank the reviewer for the careful evaluation of our manuscript and for the thoughtful summary of the scope and goals of this work. We appreciate the reviewer's recognition that CIPHER provides an integrated framework for learning assay-aware gene-to-signature encodings under explicit experimental constraints. We also appreciate the reviewer's identification of the potential strengths of this approach, as well as the important questions it raises regarding generality, parameter sensitivity, and broader utility for discovery-oriented applications. The comments below were helpful in highlighting areas where the manuscript required clearer explanation and stronger validation, and we have revised the manuscript accordingly.

Major:
The focus on separating known annotated classes may risk prioritizing known class-distinguishing genes over those that capture continuous programs (cell cycle, stress) or rare, novel cell subtypes. If the taxonomy is noisy or less well defined like in disease or developmental contexts, the encoder may oversimplify representation and miss capability on biological discovery. Thus the resulting compressed design may be less useful for unsupervised discovery, although still with the advantage of whole tissue profiling at scale. Held-out-state experiments may quantify the risk/performance for subtype discovery compatibility.

We thank the reviewer for raising this important point. In the revised Discussion, we now state explicitly that optimizing aggregate encodings for robust assignment to known classes creates a tradeoff with unsupervised discovery of rare, novel, or continuously varying states, particularly when the reference taxonomy is incomplete or noisy. We also clarify that, despite this limitation, aggregate measurements remain highly valuable for whole-tissue profiling at scale and note held-out-state benchmarking as an important future direction.

This creates an inherent tradeoff. Encodings optimized for robust assignment to known classes may be less suitable for unsupervised discovery of rare, novel, or continuously varying states, especially when the reference taxonomy is incomplete or noisy. Nevertheless, aggregate measurements remain highly valuable when the main goal is whole-tissue profiling at scale with reliable alignment to a reference.

Current CIPHER is mainly trained on Allen Brain Atlas with stratified sampling. How does different sampling affect encoded design?

We thank the reviewer for this question. The Allen Brain Atlas reference is highly imbalanced at the subclass level, with a small number of abundant non-neuronal subclasses accounting for a disproportionately large fraction of all cells, so training on the raw distribution would have driven the design toward those dominant populations at the expense of the many rarer neuronal subclasses. We therefore used stratified sampling to ensure that the encoding design reflected the full subclass taxonomy rather than being dominated by a few highly abundant cell types, and we have clarified this rationale in the revised Methods adding the following text:

The Allen Brain Atlas subclass distribution is highly imbalanced, with a small number of abundant non-neuronal subclasses comprising a disproportionately large fraction of all cells. Without stratified sampling, optimization would be

dominated by these abundant populations and would underweight the many rarer neuronal subclasses that are central to the intended classification task.

The benchmarking of how different scRNA reference will affect CIPHER design is lacking. Similarly, the reliability in typical cross-study deployments is unclear. Controlled stress tests, such as training on one dataset/region and testing on another, or training on healthy tissue and testing on perturbed, are necessary to establish robustness against batch effects or biological shifts.

We agree that robustness to reference shift is important and was not sufficiently addressed in the original submission. To provide a controlled stress test, we exploited the separation between 10x v2 and v3 chemistries within the Allen whole-brain reference and evaluated all four train-test combinations by training CIPHER on one chemistry and testing on held-out cells from either the same or the alternate chemistry. This analysis showed that performance was relatively stable to moderate loss-weight perturbations, but decreased substantially under cross-chemistry transfer, indicating that CIPHER is more sensitive to systematic shifts in the reference data than to precise tuning of the objective. We have now added this analysis as a new main-text figure panel, described it in the Methods and Results, and incorporated its implications into the Discussion as an explicit limitation of reference-based design. The new text in the Discussion section is:

A controlled cross-chemistry stress test using the 10x v2 and v3 partitions of the Allen reference further showed that transfer across chemistries was substantially weaker than within-chemistry performance, indicating that CIPHER is sensitive not only to the biological representativeness of the reference atlas but also to technical shifts between training and deployment data. This limitation is consistent with the broader problem of batch effects in scRNA-seq, where differences in chemistry, protocol, and dataset composition can distort alignment, label transfer, and downstream classification even when the underlying cell populations are similar \cite{Luecken2022Benchmarking}.

The authors comprehensively tested the effect from different loss terms of user defined constraints on multi-objective loss training. It will be useful to describe how these constraints shall be selected generally, and whether they will match the range in real spatial data, such as brightness bits. Is the objective convex?

The objective is not convex as stated in the manuscript

Providing benchmarking/approach about how to weight different loss terms is necessary for reasonable hyperparameter choice.

We now include a new figure (Figure 5) that performed sensitivity analysis for loss function weights. The figure shows that the three main evaluation metrics are not very sensitive to changes in the loss weights. Saying that, the CIPHER code does use specific targets in the optimization, which are important as shown in Figure 4, especially brightness. To provide more guidelines on how to chose these targets, we added a section to the Method (Selection of Target Values) that explains in detail the conversion of experimental poarameters related to the microscope and data acquisition setup and how they are translated into the loss targets used here. We also added the following text to the discussion section to provide more high-level guidelines that would supplement the details provided in the Methods section:

These results also suggest a practical interpretation for applying CIPHER across tissues and assay regimes. If designs achieve good nominal accuracy but poor robustness, increase separation and dynamic-range targets rather than decoder complexity. If bit intensities are systematically weak, increase brightness and probe-budget targets jointly. If performance saturates early with excess measurement capacity, reduce the number of bits or relax brightness targets.

Fig6a shows a CIPHER encoded annotation of spatial data; a trend of less distinguishable cortex layers, and misannotation of non-DG cell assignment at DG triangle seems to exist.

We agree that no annotation is error free, and this limitation is particularly relevant for compressed aggregate measurements. We therefore benchmarked the experimental CIPHER assignments against matched MERFISH reference data from the same anatomical region in Figure 6 and quantified concordance by greedy spatial pairing and expression correlation . These comparisons provide an empirical assessment of annotation quality and help place visible local discrepancies in the proper context.

For tissue generalizability, the spatial result and cell type annotation of mouse lymph node shall be shown.

While we agree that the addition of additional experimental data would support this work, we are unable to do that and consider this to be beyond the scope of the current work.

The method's reliability in typical cross-study deployments is unclear. Controlled stress tests, such as training on one dataset/region and testing on another, or training on healthy tissue and testing on perturbed, are necessary to establish robustness against batch effects or biological shifts.

We agree that the original manuscript did not adequately address reliability under reference shift. To address this point, we added a controlled stress test exploiting the separation between 10x v2 and v3 chemistries within the Allen whole-brain reference, and evaluated all four train-test combinations by training on one chemistry and testing on held-out cells from either the same or the alternate chemistry. This analysis showed that within-chemistry performance remained substantially higher than cross-chemistry transfer, indicating that CIPHER is sensitive to batch- or chemistry-dependent shifts in the reference data. We now present this result in the main text and discuss it explicitly as a limitation of reference-based design, emphasizing that matched or harmonized references are preferable for deployment.

Minor
According to common practice Fig1 could mark 'encoder' 'decoder'.

Fixed.

Fig.6A could include legend to show cell type. 6D: high resolution zoom of panel C? the region shall be highlighted.

Added zoom highlighting. Adding a color map of 334 cell types is unrealistic. We use colors that match the BICCN reference Atlas and now reference it in the figure legend.

Reviewer #3: The manuscript describes CIPHER, a new computational method to define aggegrate signatures for identifying distinct cell-types in spatial transcriptomics. It uses a matrix decomposition-like encoder with a traditional decoder with various constraint-based loss function. The approach is interesting, but performance was only marginally better than existing alternatives. In addition, the lack of methodological details, poor figures, undefined performance criteria, and lack of demonstrated biological plausibility and experimentally tractability of the method's output lowers the confidence in the presented method and results. The code is available, and I was able to successfully install it.

We thank the reviewer for the careful evaluation of our manuscript and for taking the time to install the code and assess the method directly. We appreciate the reviewer's recognition that the overall approach is of interest, and we also appreciate the frank assessment of the manuscript's weaknesses in its previous form. In particular, the reviewer's comments highlighted the need for clearer methodological description, improved figure presentation, more explicit definitions of the performance criteria, and stronger support for the biological plausibility and experimental tractability of the designed outputs. These comments were helpful in identifying the areas that most required revision, and we have substantially revised the manuscript accordingly.

"Gene expression matrices were normalized to counts per 100,000 (CP100k) by dividing each cell by its total library 63 size and multiplying by 10^5"

- Most scRNAseq datasets have fewer than 10,000 UMIs/cell, normalizing to 100,000 would greatly exaggerate the differences between 0 and 1 UMI detected. The authors should justify their choice of normalization strategy.

Expression values were not rounded after library-size normalization. In the preprocessing code, raw counts are multiplied by a continuous scaling factor equal to 100,000 divided by each cell's library size, and the resulting matrices are stored and loaded as floating-point tensors. Thus, CP100k does not discretize or amplify 1-UMI observations beyond the usual library-size normalization; relative to CP10k, it differs only by a global factor of 10. We used CP100k as a numerical

convention so that projected bit intensities and brightness targets fall in a convenient range for the experimentally motivated loss terms. We added clarification for this in the Methods section:

Gene expression values were normalized to CP100k by library-size scaling. Since normalized values were not rounded, this differs from CP10k only by a global factor of 10 and serves mainly to place projected bit intensities and brightness targets in a convenient numerical range.

- I could not find the equations for the specification of the loss function. The loss function is crucial to the model performance and reproducibility of the work they should be included in the manuscript.

We appologize for missing those out in the prior version of the manuscript. All loss functions are now fully defined in the Methods section.

- Is the simulated noise realististic for aggregate transcriptomic experiments? The authors should show some evidence that this is a reasonable assumption. Noise is often related to the magnitude of the signal itself, it is unintuitive to have fixed noise, so the authors should justify the chosen noise model.

We thank the reviewer for their feedback on the noise model. We replaced the noise model with multiplicative one as explained in the Methods section Noise Injection and Robustness. We updated Figure 5 analysis based on this model. We kept the numerical magnitude of the noise similar to previous noise model version using calibration.

- All evaluation metrics need to be rigourously defined. Otherwise the results are uninterpretable. What is "1" vs "2" for cluster separabiltiy? Are either of those actually good scores?

All evaluation metrics are now defined in the Evaluation Metrics Methods section.

- The figures need much improvement a lot of text is overlapping there's random text in many places uneven spacing in diagrams, captions have both Fig1 and Figure 1. Figure 1 should be broken up into different panels to ease explanation in the caption. Letter sub panels in figure captions don't match the figures themselves. Figure 6 lacks scale bars. Figure 7c lacks a colour scheme. Figure 7d lacks annotation, it just looks like a blob, how does it support the conclusion that the cell types are separable???

Thank you for the feedback on the figure, they have been fully reworked to address these points.

- The examples of application of CIPHER to real spatial data would be greatly improved if the authors actually listed the selected probes for each bit to convince the reader the selected probes do match biologically plausible cell-type defining markers and that the number of probes is reasonable for the ATLAS or related aggregate signature systems.

We agree that the biological plausibility of the learned aggregate features should be shown more explicitly. To address this, we added a supplementary table reporting Gene Ontology enrichment for the genes assigned to each CIPHER bit, where each bit was analyzed using the set of genes with non-zero weight in the corresponding encoding column. This provides a compact summary showing that many bits are associated with interpretable biological programs, while the full bit compositions and probe allocations are also reported for reference.

To assess biological interpretability of the learned encodings, we examined the genes assigned to each CIPHER bit and summarized their associated functions by gene ontology enrichment analysis. Across bits, enriched terms were consistent with expected neuronal, glial, and regional transcriptional programs rather than arbitrary gene mixtures, supporting the biological plausibility of the learned aggregate signatures. The full gene and probe composition of each bit, together with enrichment results, is provided in Supplementary Table 1.
