# Supplementary material for: CIPHER: An end-to-end framework for designing optimized aggregated spatial transcriptomics experiments
Source: PLoS Comput Biol. 2026 Jun 4;22(6):e1014362. doi: 10.1371/journal.pcbi.1014362 (PMC13252831; doi:10.1371/journal.pcbi.1014362)
Supplement: Attachment — Submitted filename: CIPHER-review.docx [file pcbi.1014362.s003.docx]

Here, Hemminger et al. proposed CIPHER, an end-to-end neural network framework that jointly optimizes probe encoding design and cell-type decoding for aggregate spatial transcriptomics. The key innovation is incorporating experimental constraints (probe budget, signal brightness, dynamic range) directly into the loss function, enabling end-to-end optimization that balances classification accuracy with measurement robustness. Using the spatial transcriptomics datasets under three contexts, the authors demonstrated that accuracy-only optimization yields encodings that are fragile under realistic noise, while CIPHER's multi-objective approach achieves robust cell-type separation with minimal accuracy trade-off. The framework addresses an important gap between in silico optimization and in situ measurement feasibility. I have the following major concerns that need to be revised before I will be able to recommend the manuscript for publication:

**Major Comments:**

1. CIPHER is a supervised learning method utilizing cell-type labels, whereas DPNMF is unsupervised. Comparing exclusively against an unsupervised baseline is insufficient to demonstrate CIPHER's advantages. The authors should include comparisons with other supervised approaches, such as linear discriminant analysis on marker genes, supervised PCA, or other task-aware dimensionality reduction methods.
2. The loss function weights are presented as fixed defaults without robustness analysis. Please provide sensitivity analysis showing how performance varies across weight configurations, and offer practical guidance for users on how to adjust hyperparameters for different tissues or experimental setups.
3. The noise model is oversimplified. Training uses only uniform additive noise [0, 1e4], yet real experiments exhibit gene-specific probe efficiency variations, spatially structured background fluorescence, and even channel crosstalk. The authors should either fit a noise model from real MERFISH data, or at minimum incorporate gene-specific multiplicative noise to better approximate experimental conditions.

**Minor Comments:**

1. Definitions of evaluation metrics (Accuracy, Separation, and Dynamic Fold) are incomplete. Please provide explicit mathematical definitions in the corresponding section.
2. Figure 5 requires clarification: (a) correct "seperation" to "separation" in axis labels; (b) specify axis labels as "cell-type separation" (y-axis) and "cell-type count" (x-axis) for better understanding of performance; (c) explain what the numerical values represent in panel 5b.
3. In Figure 6, indicate which region of panel 6c corresponds to the zoomed view in panel 6d.
4. Figure 7 captions do not match the panel descriptions in the main text. Please reconcile.
