# Supplementary material for: CIPHER: An end-to-end framework for designing optimized aggregated spatial transcriptomics experiments
Source: PLoS Comput Biol. 2026 Jun 4;22(6):e1014362. doi: 10.1371/journal.pcbi.1014362 (PMC13252831; doi:10.1371/journal.pcbi.1014362)
Supplement: Peer review history

# Peer review: Decision Letter 0

**DOI:** 10.1371/journal.pcbi.1014362.r001

**Authors:** Lin Wan

3 Mar 2026

PCOMPBIOL-D-26-00141

CIPHER: An end-to-end framework for designing optimized aggregated spatial transcriptomics experiments

PLOS Computational Biology

Dear Dr. Wollman,

Thank you for submitting your manuscript to PLOS Computational Biology. After careful consideration, we feel that it has merit but does not fully meet PLOS Computational Biology's publication criteria as it currently stands. Therefore, we invite you to submit a revised version of the manuscript that addresses the points raised during the review process.

Please submit your revised manuscript by May 03 2026 11:59PM. If you will need more time than this to complete your revisions, please reply to this message or contact the journal office at ploscompbiol@plos.org. When you're ready to submit your revision, log on to https://www.editorialmanager.com/pcompbiol/ and select the 'Submissions Needing Revision' folder to locate your manuscript file.

Please include the following items when submitting your revised manuscript:

* A letter that responds to each point raised by the editor and reviewer(s). You should upload this letter as a separate file labeled 'Response to Reviewers'. This file does not need to include responses to formatting updates and technical items listed in the 'Journal Requirements' section below.

* A marked-up copy of your manuscript that highlights changes made to the original version. You should upload this as a separate file labeled 'Revised Manuscript with Track Changes'.

* An unmarked version of your revised paper without tracked changes. You should upload this as a separate file labeled 'Manuscript'.

If you would like to make changes to your financial disclosure, competing interests statement, or data availability statement, please make these updates within the submission form at the time of resubmission. Guidelines for resubmitting your figure files are available below the reviewer comments at the end of this letter

We look forward to receiving your revised manuscript.

Kind regards,

Lin Wan, Ph.D.

Academic Editor

PLOS Computational Biology

Pedro Mendes

Section Editor

PLOS Computational Biology

**Additional Editor Comments :**

All three reviewers find the core idea promising, but agree that the current presentation and validation are not yet sufficiently convincing. They request stronger and fairer benchmarking—particularly the inclusion of appropriate supervised baselines rather than relying mainly on comparisons to an unsupervised method—along with a clearer and more complete methodological description (including the full loss-function equations, precise definitions of all evaluation metrics, and justification of preprocessing choices such as CP100k normalization). They also ask for more thorough robustness analyses, including sensitivity to loss weights and other hyperparameters with practical guidance for tuning, and the adoption or justification of more realistic noise models.

**Journal Requirements:**

1) Please ensure that the CRediT author contributions listed for every co-author are completed accurately and in full.

At this stage, the following Authors/Authors require contributions: Zachery Hemminger, Haley De Ocampo, Fangming Xie, Zhiqian Zhai, Jingyi Jessica Li, and Roy Wollman. Please ensure that the full contributions of each author are acknowledged in the "Add/Edit/Remove Authors" section of our submission form.

The list of CRediT author contributions may be found here: https://journals.plos.org/ploscompbiol/s/authorship#loc-author-contributions

2) We ask that a manuscript source file is provided at Revision. Please upload your manuscript file as a .doc, .docx, .rtf or .tex. If you are providing a .tex file, please upload it under the item type u2018LaTeX Source Fileu2019 and leave your .pdf version as the item type u2018Manuscriptu2019.

3) Please upload all main figures as separate Figure files in .tif or .eps format. For more information about how to convert and format your figure files please see our guidelines:

https://journals.plos.org/ploscompbiol/s/figures

4) Please amend your detailed Financial Disclosure statement. This is published with the article. It must therefore be completed in full sentences and contain the exact wording you wish to be published.

1) State the initials, alongside each funding source, of each author to receive each grant. For example: "This work was supported by the National Institutes of Health (####### to AM; ###### to CJ) and the National Science Foundation (###### to AM)."

2) State what role the funders took in the study. If the funders had no role in your study, please state: "The funders had no role in study design, data collection and analysis, decision to publish, or preparation of the manuscript."

3) If any authors received a salary from any of your funders, please state which authors and which funders.

5) Please provide a completed 'Competing Interests' statement, including any COIs declared by your co-authors. If you have no competing interests to declare, please state "The authors have declared that no competing interests exist". Otherwise please declare all competing interests beginning with the statement "I have read the journal's policy and the authors of this manuscript have the following competing interests:"

Note:If the reviewer comments include a recommendation to cite specific previously published works, please review and evaluate these publications to determine whether they are relevant and should be cited. There is no requirement to cite these works unless the editor has indicated otherwise.

**Reviewers' comments:**

Reviewer's Responses to Questions

**Comments to the Authors:**

**Please note that one review is uploaded as an attachment.**

Reviewer #1: Yes

Reviewer #2: The authors described CIPHER, which proposes an integrative end-to-end framework to compress spatial transcriptomic data by learning an assay-aware gene-to-signature encoding. The model incorporates realistic constraints (e.g., probe budget, noise, dynamic range) directly into the loss function with multiple terms to optimize together. This aims to produce compressed measurements that are both informative for cell-identity separation and feasible to measure experimentally. While promising, the current formulation may be specialized per experimental design/tissue and sensitive to loss term contribution, potentially limiting its broader utility for discovery-oriented biology.

Major:

The focus on separating known annotated classes may risk prioritizing known class-distinguishing genes over those that capture continuous programs (cell cycle, stress) or rare, novel cell subtypes. If the taxonomy is noisy or less well defined like in disease or developmental contexts, the encoder may oversimplify representation and miss capability on biological discovery. Thus the resulting compressed design may be less useful for unsupervised discovery, although still with the advantage of whole tissue profiling at scale. Held-out-state experiments may quantify the risk/performance for subtype discovery compatibility.

Current CIPHER is mainly trained on Allen Brain Atlas with stratified sampling. How does different sampling affect encoded design? The benchmarking of how different scRNA reference will affect CIPHER design is lacking.

Similarly, the reliability in typical cross-study deployments is unclear. Controlled stress tests, such as training on one dataset/region and testing on another, or training on healthy tissue and testing on perturbed, are necessary to establish robustness against batch effects or biological shifts.

The authors comprehensively tested the effect from different loss terms of user defined constraints on multi-objective loss training. It will be useful to describe how these constraints shall be selected generally, and whether they will match the range in real spatial data, such as brightness bits. Is the objective convex?

Providing benchmarking/approach about how to weight different loss terms is necessary for reasonable hyperparameter choice.

Fig6a shows a CIPHER encoded annotation of spatial data; a trend of less distinguishable cortex layers, and misannotation of non-DG cell assignment at DG triangle seems to exist.

For tissue generalizability, the spatial result and cell type annotation of mouse lymph node shall be shown.

The method's reliability in typical cross-study deployments is unclear. Controlled stress tests, such as training on one dataset/region and testing on another, or training on healthy tissue and testing on perturbed, are necessary to establish robustness against batch effects or biological shifts.

Minor

According to common practice Fig1 could mark ‘encoder’ ‘decoder’.

Fig.6A could include legend to show cell type. 6D: high resolution zoom of panel C? the region shall be highlighted.

Reviewer #3: The manuscript describes CIPHER, a new computational method to define aggegrate signatures for identifying distinct cell-types in spatial transcriptomics. It uses a matrix decomposition-like encoder with a traditional decoder with various constraint-based loss function. The approach is interesting, but performance was only marginally better than existing alternatives. In addition, the lack of methodological details, poor figures, undefined performance criteria, and lack of demonstrated biological plausibility and experimentally tractability of the method's output lowers the confidence in the presented method and results. The code is available, and I was able to successfully install it.

"Gene expression matrices were normalized to counts per 100,000 (CP100k) by dividing each cell by its total library 63

size and multiplying by 10^5"

- Most scRNAseq datasets have fewer than 10,000 UMIs/cell, normalizing to 100,000 would greatly exaggerate the differences between 0 and 1 UMI detected. The authors should justify their choice of normalization strategy.

- I could not find the equations for the specification of the loss function. The loss function is crucial to the model performance and reproducibility of the work they should be included in the manuscript.

- Is the simulated noise realististic for aggregate transcriptomic experiments? The authors should show some evidence that this is a reasonable assumption. Noise is often related to the magnitude of the signal itself, it is unintuitive to have fixed noise, so the authors should justify the chosen noise model.

- All evaluation metrics need to be rigourously defined. Otherwise the results are uninterpretable. What is "1" vs "2" for cluster separabiltiy? Are either of those actually good scores?

- The figures need much improvement a lot of text is overlapping there's random text in many places uneven spacing in diagrams, captions have both Fig1 and Figure 1. Figure 1 should be broken up into different panels to ease explanation in the caption. Letter sub panels in figure captions don't match the figures themselves. Figure 6 lacks scale bars. Figure 7c lacks a colour scheme. Figure 7d lacks annotation, it just looks like a blob, how does it support the conclusion that the cell types are separable???

- The examples of application of CIPHER to real spatial data would be greatly improved if the authors actually listed the selected probes for each bit to convince the reader the selected probes do match biologically plausible cell-type defining markers and that the number of probes is reasonable for the ATLAS or related aggregate signature systems.

**********

**Have the authors made all data and (if applicable) computational code underlying the findings in their manuscript fully available?**

The PLOS Data policy requires authors to make all data and code underlying the findings described in their manuscript fully available without restriction, with rare exception (please refer to the Data Availability Statement in the manuscript PDF file). The data and code should be provided as part of the manuscript or its supporting information, or deposited to a public repository. For example, in addition to summary statistics, the data points behind means, medians and variance measures should be available. If there are restrictions on publicly sharing data or code —e.g. participant privacy or use of data from a third party—those must be specified.

Reviewer #1: None

Reviewer #2: Yes

Reviewer #3: Yes

**********

PLOS authors have the option to publish the peer review history of their article (what does this mean?). If published, this will include your full peer review and any attached files.

If you choose “no”, your identity will remain anonymous but your review may still be made public.

**Do you want your identity to be public for this peer review?** For information about this choice, including consent withdrawal, please see our Privacy Policy.

Reviewer #1: No

Reviewer #2: No

Reviewer #3: No

[NOTE: If reviewer comments were submitted as an attachment file, they will be attached to this email and accessible via the submission site. Please log into your account, locate the manuscript record, and check for the action link "View Attachments". If this link does not appear, there are no attachment files.]

**Figure resubmission:**

While revising your submission, we strongly recommend that you use PLOS’s NAAS tool (https://ngplosjournals.pagemajik.ai/artanalysis) to test your figure files. NAAS can convert your figure files to the TIFF file type and meet basic requirements (such as print size, resolution), or provide you with a report on issues that do not meet our requirements and that NAAS cannot fix.

After uploading your figures to PLOS’s NAAS tool - https://ngplosjournals.pagemajik.ai/artanalysis, NAAS will process the files provided and display the results in the "Uploaded Files" section of the page as the processing is complete. If the uploaded figures meet our requirements (or NAAS is able to fix the files to meet our requirements), the figure will be marked as "fixed" above. If NAAS is unable to fix the files, a red "failed" label will appear above. When NAAS has confirmed that the figure files meet our requirements, please download the file via the download option, and include these NAAS processed figure files when submitting your revised manuscript.

**Reproducibility:**

To enhance the reproducibility of your results, we recommend that authors of applicable studies deposit laboratory protocols in protocols.io, where a protocol can be assigned its own identifier (DOI) such that it can be cited independently in the future. Additionally, PLOS ONE offers an option to publish peer-reviewed clinical study protocols. Read more information on sharing protocols at https://plos.org/protocols?utm_medium=editorial-email&utm_source=authorletters&utm_campaign=protocols

Attachment Submitted filename: CIPHER-review.docx

# Author comment: Author response to Decision Letter 1

**DOI:** 10.1371/journal.pcbi.1014362.r002

23 Apr 2026

Attachment Submitted filename: Response to reviewers.pdf

# Peer review: Decision Letter 1

**DOI:** 10.1371/journal.pcbi.1014362.r003

**Authors:** Lin Wan

26 May 2026

Dear Associate Professor Wollman,

We are pleased to inform you that your manuscript 'CIPHER: An end-to-end framework for designing optimized aggregated spatial transcriptomics experiments' has been provisionally accepted for publication in PLOS Computational Biology.

Before your manuscript can be formally accepted you will need to complete some formatting changes, which you will receive in a follow up email. A member of our team will be in touch with a set of requests.

Please note that your manuscript will not be scheduled for publication until you have made the required changes, so a swift response is appreciated.

IMPORTANT: The editorial review process is now complete. PLOS will only permit corrections to spelling, formatting or significant scientific errors from this point onwards. Requests for major changes, or any which affect the scientific understanding of your work, will cause delays to the publication date of your manuscript.

Should you, your institution's press office or the journal office choose to press release your paper, you will automatically be opted out of early publication. We ask that you notify us now if you or your institution is planning to press release the article. All press must be co-ordinated with PLOS.

Thank you again for supporting Open Access publishing; we are looking forward to publishing your work in PLOS Computational Biology.

Best regards,

Lin Wan, Ph.D.

Academic Editor

PLOS Computational Biology

Pedro Mendes

Section Editor

PLOS Computational Biology

***********************************************************

Please address Reviewer 2's minor comments.

Reviewer's Responses to Questions

**Comments to the Authors:**

**Please note here if the review is uploaded as an attachment.**

Reviewer #1: All concerns have been adequately addressed. I recommend acceptance.

Reviewer #2: The authors have clarified previous comments and added analyses such as benchmarking chemistry analyses to show robustness, etc. Regarding demonstrating tissue generalizability, Fig.8C analyzed mouse development dataset to show "learned latent vectors (P) organized by developmental lineage" providing evidence for "structured assignment of progenitor and neuronal populations to specific bits.". This demonstrated cell type separability and trajectory recovery. Similarly in the lymph node dataset analysis that authors did as mentioned in methods and Fig.8D, more interpretations/demonstrations on how different immune cells are separable by the proposed strategy will support tissue heterogeneity.

Reviewer #3: I thank the authors for thoroughly addressing my comments and while I still would prefer more elucidation of the biology in their practical example, the manuscript is sufficiently improved for publication.

**********

**Have the authors made all data and (if applicable) computational code underlying the findings in their manuscript fully available?**

The PLOS Data policy requires authors to make all data and code underlying the findings described in their manuscript fully available without restriction, with rare exception (please refer to the Data Availability Statement in the manuscript PDF file). The data and code should be provided as part of the manuscript or its supporting information, or deposited to a public repository. For example, in addition to summary statistics, the data points behind means, medians and variance measures should be available. If there are restrictions on publicly sharing data or code —e.g. participant privacy or use of data from a third party—those must be specified.

Reviewer #1: None

Reviewer #2: Yes

Reviewer #3: Yes

**********

PLOS authors have the option to publish the peer review history of their article (what does this mean?). If published, this will include your full peer review and any attached files.

If you choose “no”, your identity will remain anonymous but your review may still be made public.

**Do you want your identity to be public for this peer review?** For information about this choice, including consent withdrawal, please see our Privacy Policy.

Reviewer #1: **Yes:** Shaokun An

Reviewer #2: No

Reviewer #3: No

# Editor report: Acceptance letter

**DOI:** 10.1371/journal.pcbi.1014362.r004

**Authors:** Lin Wan

PCOMPBIOL-D-26-00141R1

CIPHER: An end-to-end framework for designing optimized aggregated spatial transcriptomics experiments

Dear Dr Wollman,

I am pleased to inform you that your manuscript has been formally accepted for publication in PLOS Computational Biology. Your manuscript is now with our production department and you will be notified of the publication date in due course.

The corresponding author will soon be receiving a typeset proof for review, to ensure errors have not been introduced during production. Please review the PDF proof of your manuscript carefully, as this is the last chance to correct any errors. Please note that major changes, or those which affect the scientific understanding of the work, will likely cause delays to the publication date of your manuscript.

Soon after your final files are uploaded, unless you have opted out, the early version of your manuscript will be published online. The date of the early version will be your article's publication date. The final article will be published to the same URL, and all versions of the paper will be accessible to readers.

For Research, Software, and Methods articles, you will receive an invoice from PLOS for your publication fee after your manuscript has reached the completed accept phase. If you receive an email requesting payment before acceptance or for any other service, this may be a phishing scheme. Learn how to identify phishing emails and protect your accounts at https://explore.plos.org/phishing.

Thank you again for supporting PLOS Computational Biology and open-access publishing. We are looking forward to publishing your work!

With kind regards,

Zsofia Freund

PLOS Computational Biology | Carlyle House, Carlyle Road, Cambridge CB4 3DN | United Kingdom ploscompbiol@plos.org | Phone +44 (0) 1223-442824 | ploscompbiol.org | @PLOSCompBiol
